# The prevalence, incidence and prevention of *Plasmodium falciparum* infections in forest rangers in Bu Gia Map National Park, Binh Phuoc province, Vietnam: a pilot study

**DOI:** 10.1186/s12936-017-2091-6

**Published:** 2017-11-06

**Authors:** Do Hung Son, Nguyen Thuy-Nhien, Lorenz von Seidlein, Truong Le Phuc-Nhi, Ngo Thi Phu, Nguyen Thi Kim Tuyen, Nguyen Huyen Tran, Nguyen Van Dung, Bui Van Quan, Nicholas P. J. Day, Arjen M. Dondorp, Nicholas J. White, Guy E. Thwaites, Tran Tinh Hien

**Affiliations:** 1 Oxford University Clinical Research Unit, Wellcome Trust Major Overseas Programme, Ho Chi Minh City, Vietnam; 2 Mahidol Oxford Tropical Medicine Research Unit, Faculty of Tropical Medicine, Mahidol University, Bangkok, Thailand; 3 Nuffield Department of Medicine, Centre for Tropical Medicine and Global Health, Churchill Hospital, Oxford, UK; 4 Binh Phuoc Malaria Prevention and Control Center, Dong Xoai, Binh Phuoc Vietnam

**Keywords:** *Plasmodium falciparum*, *Plasmodium vivax*, *An. dirus*, *An. maculatus*, *An. barbirostris*, Anti-malarial resistance, Forest, Prophylaxis, DHA, Piperaquine, Kelch K13-C580Y, Plasmepsin

## Abstract

**Background:**

Prophylaxis for high-risk populations, such as forest workers, could be one component for malaria elimination in the Greater Mekong Sub-region. A study was conducted to assess the malaria incidence in forest rangers and the feasibility of malaria prophylaxis for rangers sleeping in forest camps.

**Methods:**

Forest rangers deployed in the Bu Gia Map National Park, Vietnam were invited to participate in the study. *Plasmodium* infections were cleared using presumptive treatment, irrespective of malaria status, with a 3-day course dihydroartemisinin/piperaquine (DP) and a 14-day course of primaquine. Before returning to the forest, study participants were randomly allocated to a 3-day course of DP or placebo. Fifteen days after returning from their forest deployment the participants were tested for *Plasmodium* infections using uPCR.

**Results:**

Prior to treatment, 30 of 150 study participants (20%) were found to be infected with *Plasmodium*. Seventeen days (median) after enrolment the rangers were randomized to DP or placebo 2 days before returning to forest camps where they stayed between 2 and 20 days (median 9.5 days). One ranger in the DP-prophylaxis arm and one in the placebo arm were found to be infected with *Plasmodium falciparum* 15 days (median) after returning from the forest. The evaluable *P. falciparum* isolates had molecular markers indicating resistance to artemisinins (K13-C580Y) and piperaquine (plasmepsin), but none had multiple copies of pfmdr1 associated with mefloquine resistance.

**Conclusion:**

Anti-malarial prophylaxis in forest rangers is feasible. The findings of the study highlight the threat of multidrug-resistant malaria.

*Trial registration* NCT02788864

**Electronic supplementary material:**

The online version of this article (10.1186/s12936-017-2091-6) contains supplementary material, which is available to authorized users.

## Background

Falciparum and vivax malaria cases have dropped in Vietnam from over one million resulting in 4500 deaths in 1991 to 19,252 confirmed malaria cases with 25 recorded deaths in 2015 [1]. The goal of malaria elimination has been endorsed by the Vietnamese Government, but this goal is jeopardized by the emergence and spread of *Plasmodium falciparum* resistance to artemisinins and partner drugs [2–5]. Several new anti-malarial drugs are in development but the availability of a new first line drug is not expected within this decade [6]. Accelerated elimination before the current arsenal of artemisinin combination therapy loses efficacy is probably the only way to stop the spread of anti-malarial resistance.

A large proportion of malaria transmission in Vietnam occurs in forested areas, which serve as perpetual sources of transmission [7–11]. The population attributable fraction of malaria cases acquired by forest-related activities has been estimated as high as 53% [12]. Malaria epidemiology is compounded by poverty, a risk factor in Vietnam for malaria like many other malaria-endemic countries [10]. Vietnamese ethnic minorities carry a disproportionately large share of the malaria burden related to their remote settlements and the need to make a living through forest-related activities [10].

Rural villagers tend to refer to the land surrounding their village as forest. Forest-related activities can range from hunting in deep primary forest to work in plantations and rice paddies. Forest-related work tends to be seasonal, does not follow a regular schedule, and is difficult for outsiders to predict. Some villagers spend months on farms miles away from their villages while others tend to return at nightfall to their homes in their villages. Some villagers are accompanied by their families while other families remain in their villages and only men work in the forest for extended periods. One unifying feature of forest work is the basic character of overnight accommodation [13] and exposure to the *Anopheles* vectors (e.g. *Anopheles dirus*), which tend to bite outdoors and during the day. Insecticide-treated bed nets have a high protective efficacy against nocturnal, indoor malaria transmission [14] but are less protective against daytime, outdoor-biting vectors like *An. dirus*. The improvised housing of forest workers is frequently poorly suited to hanging bed nets [15].

In Binh Phuoc province, forest rangers are considered a high-risk population due to their work-related exposure. 150 forest rangers are permanently employed and twice that number are intermittently employed as part-time rangers by Bu Gia Map National Park. The self-reported malaria incidence in this population is very high. Up to 80% of forest rangers state they had malaria in the previous year but no accurate incidence estimates exist. Strategies are needed to protect these forest rangers who could be an ongoing source of malaria transmission. The purpose of the study was to: (1) measure the malaria incidence in forest rangers to determine the number of participants required in future studies to estimate the efficacy of anti-malarial prophylaxis in forest rangers; and, (2) assess the feasibility of malaria prophylaxis for rangers based in forest camps.

## Methods

### Study site

**Fig. 1:**
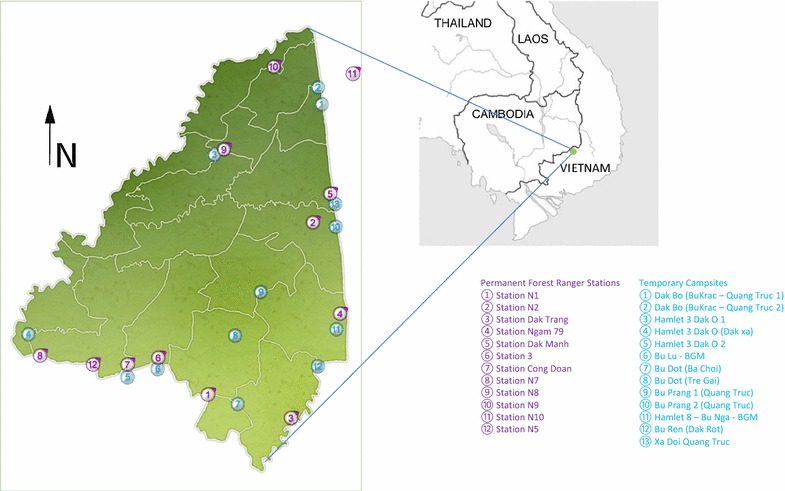
Map of National Park Bu Gia, Binh Phuoc Province, Vietnam, with forest ranger stations and campsites

Bu Gia Map National Park is dedicated to the conservation of rare and precious species of Vietnamese fauna and flora [16]. The park encompasses 26,032 ha (260 km^2^) located in an area overlapping Binh Phuoc and Dak Nong Province (Fig. 1). The park is part of the South-Central Highlands; the highest elevation is 700 m above sea level and enjoys a year-round, hot-humid, tropical climate. Entry in the park requires a permit from the park service/Ministry of Forestry. The forest and its wildlife are protected by approximately 150 permanent and 300 short-time forest rangers. The permanent rangers stay in 12 stations dispersed throughout the park, each of which accommodates 3–6 staff members. The stations are wood constructions elevated from the ground with one or two floors. Permanent rangers stay for several consecutive months in the forest. They are supported by part-time rangers recruited from hamlets surrounding the park. The short-term rangers stay in 13 camp sites some of which adjacent to the permanent stations. The accommodation of the short-term rangers is more temporary, tents and basic wooden ground floor structures. The short-time rangers spend between 2 and 20 days in the forest before returning home. All rangers tend to sleep in hammocks with or without bed nets. Malaria transmission occurs throughout the year and is slightly increased from September to March.

### Participants

Male forest rangers over 18 years of age were invited to participate in the study. Rangers who consented were enrolled at Bu Gia Map Health Station and the Office of the Forest Rangers. Candidates with anaemia (Hb < 9 mg/dl), G6PD deficiency diagnosed by fluorescent spot test or a previous history of adverse reactions to the study drugs were excluded.

### Study design and procedures

Study participants were asked to provide a blood sample on enrolment to assess parasite infections using ultrasensitive (u)PCR, and to assess the presence of G6PD deficiency. All enrolled participants received a 3-day course of dihydroartemisinin (DHA)/piperaquine (7 mg/kg/day dihydroartemisinin; 55 mg/kg/day piperaquine phosphate (Arterakine©; Pharbaco Central Pharmaceutical JSC No. 1, Hanoi, Vietnam) and 14 days of 0.25 mg/kg/day primaquine (Danaphar Pharmaceutical JSC; Da Nang City, Vietnam). The DHA/piperaquine tables were split into half tablets as needed for precise dosing. The administration of study drugs was directly observed by study staff.

Two days prior to their assignment in the forest the study participants were randomized 1:1 (block size 4) using a computer-generated schedule to receive either a 3-day course of DHA/piperaquine (Arterakine©; 7 mg/kg/day dihydroartemisinin; 55 mg/kg/day piperaquine phosphate; Pharbaco Central Pharmaceutical JSC No. 1, Hanoi, Vietnam) or placebo with identical physical properties (also Pharbaco Central Pharmaceutical JSC No. 1). Study participants and staff were blinded to the identity of the study drugs.

A venous blood sample was collected from all participants on enrolment, prior to the trip to forest trip and 14 days after their return from the forest, to detect infections still incubating on return from the forest trip.

### Detection and quantification of malaria parasitaemia

Standard microscopy was performed by microscopists who had at least 5 years’ experience and/or were confirmed to be Level 2 or higher with a WHO 55 slide set. Thick and thin blood films were examined at 3 visits during the study period (base line, pre-forest and post-forest). Specimens were labelled to maintain anonymity (study number, day of visit, and date). Thick and thin blood smears were examined were stained with 10% Giemsa in pH 7.2 buffer solution for 15 min. Parasitaemia was estimated by counting the number of asexual parasites per 500 leucocytes in the thick blood film or per 1000 red blood cells on a thin film then calculated followed WHO guideline. All slides were read by two qualified microscopists independently, and the average of the two counts was taken as the parasite density. Blood smears with discordant results (differences in assessments of species, parasite density > 50%, or the presence of parasites) were re-examined by a third independent microscopist, and parasite densities were calculated by averaging the two closest counts. For rapid diagnosis, SD Bioline Malaria Ag *Pf*/*Pv* RDTs were used (SD Standard Diagnostics, Gyeonggi-do, Republic of Korea).

Sub-microscopic parasitaemia was detected by high-volume ultrasensitive qPCR (uPCR) method reported recently [17]. In summary, the parasite DNA were extracted by MagNa Pure 96 Instrument automated extraction system using MagNa Pure 96 DNA and Viral NA Small Volume Kit (Roche, Switzerland) from the thawed packed red blood cells samples. Purified DNA was dehydrated and then suspended in a small volume of PCR grade water. Two µl of resuspended DNA were used as template in the qPCR reaction. The presence of malaria parasites and an estimate of the parasite numbers (genomes) in each sample were assessed by an absolute quantitative real-time PCR (qPCR) method (Roche, Switzerland). The 18S rRNA-targeting primers and hydrolysis probes used in the assay have been validated and are highly specific for *Plasmodium* species [18]. The lower limit of accurate quantitation of this method is 22 parasites/ml of whole blood [19]. For samples where the uPCR was positive an attempt was made to determine the *Plasmodium* species present using qPCR protocols specific to *P. falciparum* (18s rRNA) and *Plasmodium vivax* (18s rRNA) described previously [18, 20–22]. Samples for which there was insufficient DNA to do this, or where no amplification was obtained in this step were reported as being of indeterminate species (*Plasmodium* spp.). uPCR results became available after study completion and did not trigger the treatment of study participants.

### Anti-malarial drug resistance markers detection

K13 gene amplification and sequencing K13 encoding gene was amplified by nested PCR then genotyped by capillary sequencing as described previously [23]. The K13 sequences were assembled by ContigExpress and aligned with the K13 gene sequence of 3D7 clone (PF3D7_1343700) using MEGA 5.0 to identify any SNPs present. Piperaquine resistance marker testing DNA samples were also sequenced to detect mutation at position 415 in putative exonuclease gene (exoE415G) following a protocol described recently [24]. Copy number of plasmepsin 2 encoding gene was detected by real time qPCR following a procedure shared by the same group [24]. Multidrug resistance marker testing copy number of pfmdr1 gene were detected by real time qPCR following a procedure published previously [25].

### Entomology

Entomological studies using CDC light traps and human landing catches were conducted for 3 nights in camps 2 and 79 and for 4 nights in camp 8. Light traps were set up following the instructions provided by the producer [26]. A single light-trap was suspended about 1.5 m above ground near a sleeping ranger. The light traps were used from sunset (18:00) to sunrise (06:00). The traps were collected in the morning by project staff. For human landing catches, approaching mosquitoes were detected by flashlight, aspirated and placed in screened, pint-sized containers. Mosquito collections were performed from 18.00 to 06:00. The light traps were used only indoors while human landing catches were conducted indoors and outdoors. All anopheline mosquitoes caught were sorted and morphologically identified using the taxonomic keys of Rattanarithikul and Panthusiri [27–33]. PCR was used to detect *Plasmodium* infections in mosquitoes [34]. Mosquitoes other than anophelines were discarded.

### Data management and analysis

Data were recorded on case report forms and double-entered in a custom-designed Access database (Microsoft, Redmond, WA, USA). Line listings of the study participants were analysed in Excel (Microsoft, Redmond, WA, USA). Proportions were compared using Chi squared or Fisher’s exact test. Stata 14.2 was used for statistical testing (StataCorp, College Station, TX, USA).

This was the first study of malaria in the forest rangers working in Bu Gia Map National Park. Neither the duration of forest stays, nor an estimate of malaria incidence or the prevalence of subclinical *Plasmodium* infections in forest rangers has been reported prior to this study. In the absence of prior estimates of prevalence and incidence of *Plasmodium* infections in this population it was not possible to estimate the sample size required to demonstrate the protective efficacy of anti-malarials.

## Results

**Fig. 2:**
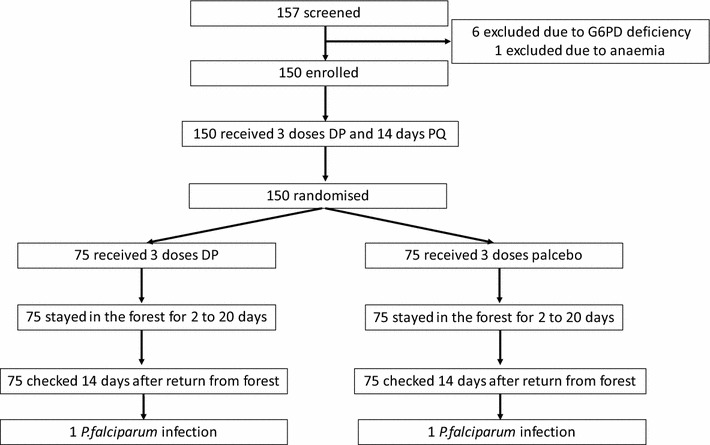
Assembly and flow of study participants

The study was conducted between May and September 2016. 157 forest rangers were screened. Six rangers were excluded due to G6PD deficiency and one due to anaemia (Fig. 2); 150 rangers were enrolled, gave a venous blood sample and received 3 days’ DP combined with 14 days’ primaquine. The study drugs were well tolerated; none of the participants reported adverse reactions to the study drugs. After a median time span of 17 days (IQR 16–17; Table 1) the 150 enrolled rangers were randomized to receive either DP or placebo and departed for the forest after completing 3 days of directly observed drug administration. The median duration of forest stay was 9.5 days (IQR 5–12 days). The median duration of study participation was 47 days (IQR 42–54). No study participant was lost to follow-up.

**Table 1:** Time spans (median and interquartile range)

	ACT	IQR	Placebo	IQR	All	IQR
Duration of last forest stay before enrolment (days)	8	4–13	8	5–14	8	5–12
Days since last forest stay	7	7–28	7	7–28	7	7–14
Days between start drug administration* and return to forest	19	19–28	19	18–20	19	18–19
Days in forest	8	5–12	10	3–10	9.5	5–10
Days between return to forest and last screening	28	23–34	28	23–34	28	23–34
Duration of study participation (days)	47	42–54	47	42–54	47	42–54

* 3 days DHA/piperaquine and 14 days PQ

### Baseline characteristics

Except for one permanent ranger, all participants were short-time forest rangers. Nearly all participants (148/150; 99%) worked as farmers when not employed as forest rangers (Table 2). There were no statistically significant differences between demographic, clinical and parasitological characteristics of participants in the artemisinin-based combination therapy (ACT) and placebo arm. The rangers had returned from the forest 14 days earlier (median; range 10–20 days) and the previous forest visit had lasted 8 days (median; IQR 5–10 days). 30/150 study participants (20%; 95% CI 13–27%) were found to be infected with *Plasmodium* at enrolment (*P. falciparum* 12/150 = 8% 95% CI 4–14%; *P. vivax* 9/150 = 6%; *P. falciparum/P. vivax* mix 2/150 = 1%; *Plasmodium* spp. 7/150 = 5%). Four of the infected participant had parasitaemia detectable by microscopy and 3 by rapid diagnostic tests (RDT). The positive results detected by RDT, microscopy and uPCR were concordant. The mean haemoglobin (Hb) of the participants was 14.7 g/dl. Eleven *P. falciparum* infections had K13 C580Y mutations in the K-13 propeller region associated with artemisinin resistance; the remaining infection could not be genotyped due to low DNA quantities. Two of the 12 *P. falciparum* infections had multiple copies of P14 associated with piperaquine resistance, 7 had single copies, and 4 samples had insufficient DNA material for testing. Eleven of the 12 *P. falciparum* infections had an exo E415G mutation associated with piperaquine resistance, one was a wild type; the other *P. falciparum* infection could not be tested due to low sample volume. None of the *P. falciparum* isolates had multiple copies of *pfmdr1.*

**Table 2:** Demographic, clinical, and parasitological characteristics of 150 study participants

	ACT	%, 95% CI	Placebo	%, 95% CI	Total	P value
n	75	50%	75	50%	150	
Baseline						
Male	75	100%	75	100%	150	1
Age (years; mean; 95% CI)	30.6	28.4–32.9	33.1	30.6–35.7	32.9	0.9
Weight (kg, mean; 95% CI)	58.3	56.5–60.2	58.4	56.5–60.4	58.4	0.9
Temperature (celsius; mean; 95% CI)	36.2	36.1–36.3	36.2	36.1–36.3	36.2	0.9
Fever (temp > 37.4)	0	0%	1	1%	1	0.3
Employment: farmer	73	97%	75	100%	148	0.4
uPCR positive	18	24%	12	16%	30	0.2
*Pf*	8	11%	4	5%	12	0.4
*Pv*	7	9%	2	3%	9	0.2
*Pf*/*Pv* mix	1	1%	1	1%	2	1
*P* spp.	2	3%	5	7%	7	0.4
RDT positive	2	3%	1	1%	3	0.6
Microscopy positive	3	4%	1	1%	4	0.3
Hb (mg/dl; mean; 95% CI)	14.6	14.2–14.9	14.7	14.3–15.0	14.6	0.7
Pre-forest entry						
Fever (temp > 37.4)	0	0%	0	0%	0	1
uPCR pos	1	1%	4	5%	5	0.4
Pf	1	1%	2	3%	3	1
Pv	0	0%	0	0%	0	na
Pspp	0	0%	2	3%	2	0.5
RDT positive	0	0%	0	0%	0	na
Microscopy positive	0	0%	0	0%	0	na

#### Pre-forest entry

Two out of 150 participants (1%) were found to be infected with *P. falciparum* after treatment and before returning to the forest (Table 3). The parasite densities of both infections were low (663, 6412/ml). Two days before returning to the forest all 150 study participants received either a 3-day course DHA/piperaquine (DP) or placebo.

**Table 3:** Key data of 3 study participants infected on enrolment and 2 participants who acquired *Plasmodium* infections during the study period

Participant	#107	#124	#006
Baseline			
Date 1	21/7/16	24/8/16	20/5/16
Species	*Pf*	*Pv*	Not infected
K13	C580Y	Not tested	
ExoE415G	E415G		
P14 CNV	Single copy		
Pfmdr1 CNV	Single copy		
Parasites/ml	22,225	1505	
Treatment 1	ACT + 14 days primaquine (15 mg/day)		
Pre-forest entry			
Date 2	7/8/16	9/10/16	6/6/16
Species	*Pf*	Not infected	*Pf*
K13	C580Y		Wild type
ExoE415G	E415G		Insufficient DNA sample for testing
P14 CNV	Multicopies		
Pfmdr1 CNV	Single copy		Single copy
Parasites/ml	6412		663
Treatment 2	ACT	ACT	Placebo
Forest days	10	5	20
Camp	Not known	Campsite^a^	Camp 8 Tram 7
15 days after return from forest			
Date 3	6/9/16	4/11/16	12/7/16
Species	Not infected	*Pf*	*Pf*
K13		C580Y	C580Y
ExoE415G		E415G	E415G
P14 CNV		Multicopies	Multicopies
Pfmdr1 CNV		Single copy	Single copy
Parasites/ml		5142	111,500

^a^Camp: “campsite” refers to an overnight campsite while the rangers are moving through the forest

#### Post-forest visit

The 150 forest rangers stayed a total of 1523 days or a median of 9.5 days (IQR 5–10) in the forest. Fifteen days after their return from the forest the 150 participants were evaluated (Table 4). One asymptomatic participant in the treatment arm and one in the control arm (each 1.3%; 95% CI 0.03–7%; P = 1) was parasitaemic after returning from the forest. The incidence of *P. falciparum* infections in this population was 479/1000/year (95% CI 12–2666). Two study participants became infected while staying in the forest (Table 3). A participant in the treatment arm (#124) was uninfected prior to forest entry and had a *P. falciparum* parasitaemia of 5142/ml after staying in the forest for 5, 26 days after DP administration. A participant in the placebo arm (#006) had a *P. falciparum* parasitaemia of 111,500/ml after spending 20 days in the forest, and 36 days after DP administration. The participant was infected with wild type isolate of *P. falciparum* (K13 wild type; Pfmdr1 CNV single copy) prior to forest entry. When returning from forest work the participant was infected with a mutant *P. falciparum* isolate (PfPailin; K13 C580Y; Exo E415G; P14 CNV multi-copy).

**Table 4:** Exposure and parasitaemia detected after returning from forest at the end of the study

	ACT	%, 95% CI (n = 75)	Placebo	%, 95% CI (n = 75)	Total	P value
Did you use a bed net?						
Every night	49	65%	50	67%	99	1.0
Some nights	16	21%	11	15%	27	0.4
No bed net use	10	13%	14	19%	24	0.5
Did you use insect-repellent coil?	18	24%	8	11%	26	0.05
How many people did you meet while in forest (mean; 95% CI)	5.0	4.5–5.5	4.8	4.6–5.1	4.9	0.7
uPCR pos—*P. falciparum*	1	1%	1	1%	2	1
RDT positive	0	0%	0	0%	0	na
Microscopy positive	0	0%	1	1%	1	1

### Entomology

Entomological studies were conducted between 18:00 and 06:00 in 3 camps for 3 nights in camps 2 and 79 and for 4 nights in camp 8. Indoor light traps caught 99 *Anopheles* mosquitoes while outdoor and indoor human landing catches caught 6 and 4 *Anopheles* mosquitoes, respectively (Additional file 1: Table S1). Ninety-two of the 109 mosquitoes were *Anopheles dirus* (84%), 15 *Anopheles maculatus* (14%), and 2 *Anopheles barbirostris* (2%). PCR found *Plasmodium*-related infections in 6 of the 92 *An. dirus* (7%) and in one of the 2 *An. barbirostris* (50%). Of the two participants found infected after returning from the forest only one stayed in a camp where mosquitoes had been collected (camp 8).

## Discussion

This is the first study investigating malaria in forest rangers and approaches to prevent *Plasmodium* infections during their stay in the forest. After clearing all *Plasmodium* infections with a schizonticidal and hypnocytocidal drug combination (DP and 14 days PQ) all but two participants had cleared *Plasmodium* infections. Nearly 3 weeks later, just before returning for their next forest rotation the participating rangers received either another course of 3 doses of DP, which can provide anti-malarial prophylaxis for up to 4 weeks, or placebo. The forest rangers stayed only for a median of 9.5 days in the forest and were screened a third time 28 days after the second anti-malarial prophylaxis had started. One participant in the placebo arm was infected and one participant who had received anti-malarial prophylaxis.

Prior to the study there were no data on the prevalence and incidence of *Plasmodium* infections in forest rangers in Bu Gia Map National Park. Using uPCR, which is more sensitive than microscopy, RDTs or qPCR on dried blood spots, the study found *P. falciparum* prevalence in forest rangers was 8% and incidence in this population was 479/1000/year (95% CI 12–2666). This compares to an incidence of 332/1000/year (95% CI 253–420) among farmers in Dak O commune, also in Binh Phuoc Province, estimated in a longitudinal study also using active surveillance with uPCR in 2014 [Nguyen et al., pers. comm.]. The study did not detect any impact of the prophylaxis; one infection was detected in both the treatment and in the placebo arm. This finding could be related to the limited sample size and number of infections. Based on the malaria incidence estimate from this study, 892 participants would have to spend 14 nights each in the forest to have sufficient power (90%) for demonstrating significant protection is afforded by DP prophylaxis (Box 1).

The entomological component of the study detected three Anopheles species. The large majority were *An. dirus* (84%), followed by *An. maculatus* (14%), and *An. barbirostris* (2%). These findings are consistent with earlier entomological studies in Binh Phuoc province. Ngo and coworkers found 17 *Anopheles* species in the province amongst which *An. dirus* was most prevalent [35]. Similarly Trung and coworkers found in 2005 that *An. dirus* was most prevalent in southern Vietnam [36]. They also found that *An. dirus* was preferentially anthropophilic, biting outdoors before 22:00. The biting rhythm and resting behaviour of *An. dirus* reduces the impact of the two most commonly employed control measures, insecticide-treated bed nets and indoor residual spraying. Imaginative interventions such as supplying forest workers with insecticide treated hammocks does not address the biting rhythm and resting behaviour and had a disappointing uptake in field studies [15, 37]. In the absence of simple, effective, and affordable vector control interventions providing forest rangers with effective anti-malarial prophylaxis seems a promising alternative approach to protect forest rangers against malaria.

The study highlights the challenges of multidrug-resistant malaria to treatment and prophylaxis in the region. Treatment failures with ACT are an increasing challenge for healthcare providers treating falciparum malaria in Vietnam [3, 5]. All *P. falciparum* isolates evaluated at baseline carried genetic markers associated with artemisinin and resistance to the partner drug piperaquine. The long terminal half-life of piperaquine affords protection against infection with susceptible *P. falciparum* isolates for up to 4 weeks [38]. The presence of two *P. falciparum* infections after the directly observed administration of a course of DP in the first and second round is cause for concern. Three of the *P. falciparum* isolates carried markers for artemisinin and piperaquine resistance. The remaining 10 *P. falciparum* infections detected at baseline with K-13 and plasmepsin markers associated with artemisinin and piperaquine resistance had cleared after a course of DP due to the remaining therapeutic efficacy of DP or immunity. None of the *P. falciparum* isolates had multiple copy numbers of pfmdr1 associated with mefloquine resistance. This finding supports the notion of an inverse susceptibility between piperaquine and mefloquine and could suggest that the therapeutic efficacy of DP can be boosted by a triple therapy adding mefloquine to DP. Such a triple regimen is currently in clinical trials in the region [39]. Adapting triple therapy for prophylaxis may require co-formulation to achieve adequate adherence.

### Box 1: Sample size required to estimate the protection afforded by antimalarial prophylaxis taken prior to says in the forest

- 2 infections acquired during 1000 nights spent in the forest.
- 25 infections would be needed to have sufficient power to show a 75% protection against infection during a forest visit. (20 infections in controls, 5 infections in participants receiving effective antimalarial prophylaxis).
- 12,500 forest nights should suffice to detect 25 infections acquired during stay in the forest.
- 892 participants would have to spent 14 nights each in the forest to demonstrate significant protection is afforded DP prophylaxis.

## Conclusions

The findings of this study illustrate the challenges in preventing malaria in forest rangers in Bu Gia Map National Park. The impact of vector control approaches is limited by the biting behaviour of the dominant vector *An. dirus* and the impact of anti-malarial prophylaxis with DP is increasingly limited by the spread of anti-malarial resistance. Two populations of forest rangers, permanent and short time overlap temporarily in the park and sustain the transmission of Plasmodium infections. Breaking this cycle will require the targeting of all rangers in the forest at the same time. A mass anti-malarial administration campaign targeting all rangers in the National Park could provide a permanent solution. DP is probably no longer appropriate for such a campaign; new drug combinations will be needed. After the interruption of malaria transmission in the National Park preventing reintroduction of *Plasmodium* infections will remain a constant challenge until malaria is eliminated from Vietnam.

## Additional file

**Additional file 1: Table S1.** Entomological data.
