# Supplementary material for: The prevalence, incidence and prevention of Plasmodium falciparum infections in forest rangers in Bu Gia Map National Park, Binh Phuoc province, Vietnam: a pilot study
Source: Malar J. 2017 Nov 6;16:444. doi: 10.1186/s12936-017-2091-6 (PMC5674731; doi:10.1186/s12936-017-2091-6)
Supplement: Supplementary file 1 — Additional file 1: Table S1. Entomological data. [file 12936_2017_2091_MOESM1_ESM.docx]

Table S1. Entomological data

| Location | Date | *Anopheles* species | Human landing Catches | | Light Trap Catches |
| --- | --- | --- | --- | --- | --- |
|  |  |  | Indoors | Outdoors | Indoors |
| Camp 8 | 24/5/2016 | *An. dirus* | 0 | 0 | 11 |
|  |  | *An. maculatus* | 0 | 0 | 6 |
|  |  | *An. barbirostris* | 0 | 0 | 0 |
|  | 25/5/2016 | *An. dirus* | 1 | 0 | 5 |
|  |  | *An. maculatus* | 0 | 0 | 3 |
|  |  | *An. barbirostris* | 0 | 0 | 0 |
|  | 26/5/2016 | *An. dirus* | 0 | 5 | 17 |
|  |  | *An. maculatus* | 0 | 0 | 2 |
|  |  | *An. barbirostris* | 0 | 0 | 2 |
|  | 27/5/2016 | *An. dirus* | 2 | 0 | 17 |
|  |  | *An. maculatus* | 0 | 0 | 1 |
|  |  | *An. barbirostris* | 0 | 0 | 0 |
| Camp 2 | 28/5/2016 | *An. dirus* | 1 | 0 | 2 |
|  |  | *An. maculatus* | 0 | 0 | 0 |
|  |  | *An. barbirostris* | 0 | 0 | 0 |
|  | 29/5/2016 | *An. dirus* | 0 | 0 | 0 |
|  |  | *An. maculatus* | 0 | 0 | 0 |
|  |  | *An. barbirostris* | 0 | 0 | 0 |
|  | 30/5/2016 | *An. dirus* | 0 | 0 | 3 |
|  |  | *An. maculatus* | 0 | 0 | 0 |
|  |  | *An. barbirostris* | 0 | 0 | 0 |
| Camp 79 | 31/5/2016 | *An. dirus* | 0 | 0 | 3 |
|  |  | *An. maculatus* | 0 | 0 | 1 |
|  |  | *An. barbirostris* | 0 | 0 | 0 |
|  | 1/6/2016 | *An. dirus* | 0 | 1 | 1 |
|  |  | *An. maculatus* | 0 | 0 | 0 |
|  |  | *An. barbirostris* | 0 | 0 | 0 |
|  | 2/6/2016 | *An. dirus* | 0 | 0 | 23 |
|  |  | *An. maculatus* | 0 | 0 | 2 |
|  |  | *An. barbirostris* | 0 | 0 | 0 |
| Total |  |  | 4 | 6 | 99 |
